# The Use of Direct Oral Anticoagulants in the Management of Venous Thromboembolism in Patients With Obesity

**DOI:** 10.7759/cureus.10006

**Published:** 2020-08-25

**Authors:** Moustafa Younis, Ahmed Elkaryoni, George W Williams, Ishaan Jakhar, Sahil Suman, Stephen Simon, Gary Salzman

**Affiliations:** 1 Internal Medicine, University of Missouri-Kansas City School of Medicine, Kansas City, USA; 2 Internal Medicine, University of Missouri, Kansas City, USA; 3 Pulmonary and Critical Care, University of Missouri, Kansas City, USA; 4 Bioinformatics, University of Missouri, Kansas City, USA; 5 Internal Medicine, University of Missouri/Truman Medical Center, Kansas City, USA

**Keywords:** direct oral anticoagulant therapy, community obesity, deep vein thrombosis (dvt), acute pulmonary embolism, venous thromboembolism

## Abstract

Introduction

The use of direct oral anticoagulants (DOACs) has gained significant traction given the lack of therapeutic monitoring and the need for anticoagulant bridging. There is a paucity of data on their effectiveness in obese patients with venous thromboembolism (VTE). Preliminary subgroup and pharmacokinetic analyses suggest reduced efficacy in those with a bodyweight >120 kg or body mass index (BMI) ≥40 kg per m^2^ and it is currently not recommended that these agents be used as first-line agents. We aimed to assess the rate of VTE recurrence in obese patients diagnosed with VTE and treated with DOAC therapy.

Methods

We utilized the Health Facts Center National Data Warehouse (Cerner) to perform a retrospective analysis of patients with VTE (acute deep venous thrombosis (DVT) or pulmonary embolism) that presented to the hospital between 2010 and 2016 and were managed with DOACs. The cohort of patients diagnosed with DVT or PE were identified using International Classification of Disease (ICD-9-CM, ICD-10-CM). Patients were divided into two groups based on their weight: 1) weight <120 kg or 2) weight>120 kg. Six-month VTE recurrence rates were recorded. Summary and univariate statistics were performed.

Results

A total of 18,147 patients with a mean (±SD) age of 62 (17) years were included; 48% (n=8732) were male. A total of 2,419 (13%) patients weighed >120 kg while the rest (N=15,728, 87%) weighed <120 kg. There were significantly more female patients weighing<120 kg (54% vs 42%, p<0.0001); otherwise, there was no significant difference in age or tobacco use between both groups (p>0.05). There was no significant difference in six-month readmission rates for VTE recurrence in patients that weighed <120 kg (34%) in comparison with patients >120 kg (36%) (p=0.08).

Conclusion

Our study suggests that the use of DOACs in obese patients is equally efficacious with similar VTE recurrence rates in comparison with non-obese patients. This study paves the way for prospective multi-institutional randomized control trials to further reinforce the safe use of such agents in this patient population.

## Introduction

Options for anticoagulation have been expanding steadily over the past few decades, providing a greater number of agents for the prevention and management of thromboembolic disease. In addition to heparins and vitamin K antagonists, anticoagulants that directly target the enzymatic activity factor Xa have been developed. The use of direct oral anticoagulants (DOACs) has gained significant traction given the lack of therapeutic monitoring, dietary restrictions, and the need for anticoagulant bridging, along with its fixed dosing providing a convenient anticoagulant alternative to vitamin K antagonists.

According to the World Health Organization (WHO) in 2016, an estimated 13% of the world's population was obese (body mass index (BMI) ≥30 kg/m^2^) and the prevalence of obesity has been persistently increasing between 2007 and 2016 [1-2]. This translates to nearly 25-million obese adults living in the US [3]. The health implications of obesity are complex. Beyond obesity being a prothrombotic state, the physiologic changes associated with obesity can affect the absorption, distribution, metabolism, and excretion of administered drugs, thereby altering their pharmacologic profiles. How to best treat the obese patient with venous thromboembolism (VTE) is still an area of active investigation. Based on a 2016 review of available literature, the International Society of Hemostasis and Thrombosis (ISTH) recommends the avoidance of DOACs in individuals with a body mass index (BMI) ≥40 kg/m^2^ or weight ≥120 kg in the management of VTE as there are limited clinical data available for patients at the extreme of weight and the available pharmacokinetic/pharmacodynamic (PK/PD) evidence that raises concerns about underdosing [4]. If DOACs are used in these patients, the ISTH guidance suggests monitoring drug-specific peak and trough levels. The ISTH recommendations apply to all DOACs, although evidence suggests that different DOACs' clinical and pharmacologic profiles may not be influenced to the same extent by weight [5].

 Ultimately, the available clinical data for the use of DOACs for the management of VTE in the obese population is limited. In this study, we aimed to add to the paucity of available data on the efficacy of DOAC use in patients with extreme obesity. Herein, we retrospectively assess the rate of VTE recurrence in patients with body weight >120 kg diagnosed with VTE and treated with DOAC therapy.

## Materials and methods

Health Facts Cerner National Data Warehouse

A retrospective review of the Health Facts Cerner National Data Warehouse was performed. Cerner Health Facts is a de-identified patient database collected from over 750 healthcare facilities across the United States. Collected as a by-product of patient care, Cerner Health Facts is a comprehensive source of de-identified, real-world data. Cerner Health Facts collects clinical records with time-stamped and sequenced information on pharmacy, laboratory, admission, and billing data from all patient care locations. Researchers can analyze detailed sets of de-identified clinical data at the patient level. Cerner Health Facts includes data on patient encounters, diagnoses, procedures, medication orders, medication administration, vital signs, laboratory tests, locations of services/patients (e.g., clinic, emergency department, intensive care unit, etc.) and hospital information, and billing. As of 2018, Cerner Health Facts contains: 1) over 65 million patients, 2) patient information from 750 healthcare facilities across the United States, 3) over 500 million encounters, 4) 4.7 billion laboratory results, 5) detailed pharmacy, laboratory, billing, and registration data as far back as 2000, and 6) 684 million orders for nearly 4,500 drugs by name and brand.

Study participants

The Health Facts Data Warehouse uses widely adopted coding systems. The cohort of patients diagnosed with deep venous thrombosis (DVT) or a pulmonary embolus (PE) between January 2016 and December 2019 was identified using International Classification of Disease codes (ICD-9-CM and ICD-10-CM; see below). We subsequently used National Drug Codes (NDC) to identify patients that were treated using DOACs. DOACs used include dabigatran (Pradaxa), rivaroxaban (Xarelto), apixaban (Eliquis), or edoxaban (Savaysa). Extreme obesity was defined as weight greater than 120 kg. Patients were then divided into two groups: >120 kg and <120 kg based on their weights. Patients not on anticoagulation, patients with missing data, and patients with encounters not within our time range were excluded. Encounters before 2010 and after 2016 were excluded from the analysis because the Health Facts architecture was updated in 2009 and the data for 2017 onwards was incomplete.

List of ICD-9 and ICD-10 codes

ICD10: I82.210 I82.220 I82.290 I82.3 I82.401 I82.402 I82.403 I82.409 I82.411 I82.412 I82.413 I82.419 I82.421 I82.422 I82.423 I82.429 I82.431 I82.432 I82.433 I82.439 I82.441 I82.442 I82.443 I82.449 I82.491 I82.492 I82.493 I82.499 I82.4Y1 I82.4Y2 I82.4Y3 I82.4Y9 I82.4Z1 I82.4Z2 I82.4Z3 I82.4Z9 I82.601 I82.602 I82.603 I82.611 I82.612 I82.613 I82.619 I82.621 I82.622 I82.623 I82.629 I82.890 I82.90 I82.A11 I82.A12 I82.A13 I82.A19 I82.B11 I82.B12 I82.B13 I82.B19 I82.C11 I82.C12 I82.C13 I82.C19 I82.811 I82.812 I82.813 I82.819 Z86.718 I26.02 I26.09 I26.92 I26.99 I27.89 Z86.711

 ICD 9: 415.0 415.1 415.11 415.13 415.19 453.4 453.40 453.41 453.42 453.82 453.83 453.84 453.85 453.86 453.87 453.89 453.9

Outcomes and data points

Our primary outcome was the rate of VTE recurrence within six months of DOAC initiation. VTE was defined as either DVT or PE. Following identification of the initial diagnosis encounter, the same ICD-9 and ICD-10 codes were used to detect any further hospital admission diagnosis secondary to DVT or PE.

Other data points collected include age, weight, sex, race, and tobacco use.

Data analysis

Data analysis was performed by experts at our institution. Categorical variables were reported as counts and percentages, normally distributed continuous variables were reported as means ±standard deviation (SD), and nonparametric continuous data were reported as medians with interquartile range (IQR). The chi-square test was used to compare categorical variables, and the student’s t-test was used to compare the continuous outcomes between the two groups for uniformly distributed variables. When data were not distributed uniformly, the Wilcoxon rank-sum test was used to compare the outcomes between the two groups. The Kaplan-Meier (KM) method was used to analyze 'time-to-event' data. All tests were two-sided with an α level set at 0.05 for statistical significance. All data analysis was performed using SAS (AS Institute, Raleigh, North Carolina), SQL, and R (R Core Team).

## Results

**Figure 1:**
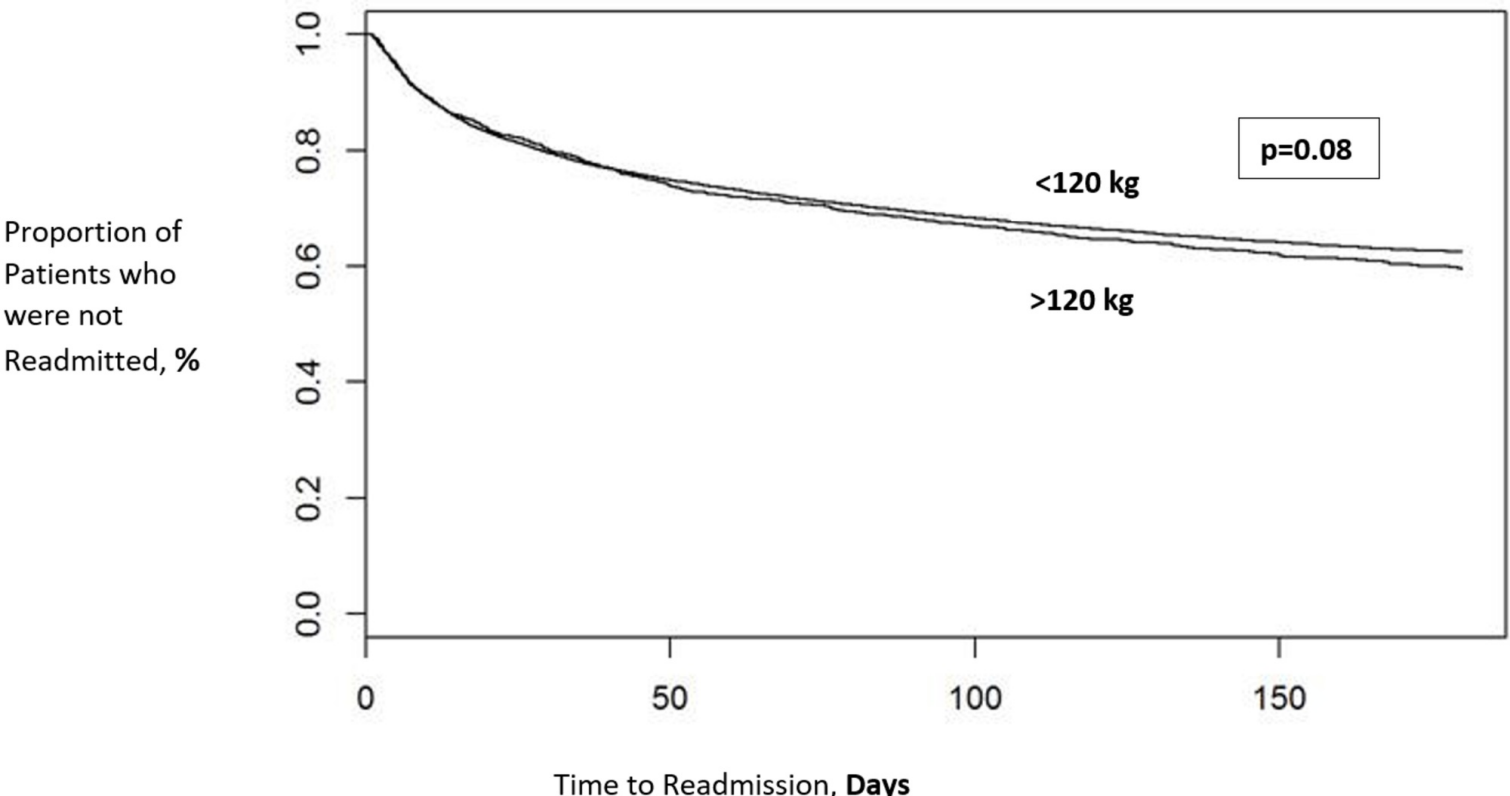
Kaplan-Meier curves comparing readmission rates in patients weighing more or less than 120 kilograms

We identified 30,124 encounters for a total of 18,147 patients who were diagnosed with DVT or PE and started on DOAC [M.1] therapy between 2010 and 2016. The mean (±SD) age was 62 (17) years; 48% (n=8732) were male. A total of 2,419 (13%) patients weighed >120 kg while the rest (N=15,728, 87%) weighed <120 kg. There were significantly more female patients weighing<120 kg (54% vs 42%, p<0.0001); otherwise, there was no significant difference in age, race, or tobacco use between both groups (p>0.05). There was no significant difference in six months readmission rates for VTE recurrence in patients that weighed <120 kg (34%) in comparison with patients >120 kg (36%) (p=0.08). See Figure 1.

## Discussion

DOACs, including the activated factor Xa inhibitors rivaroxaban, edoxaban, and apixaban, and the thrombin inhibitor dabigatran, are approved for the treatment of VTE in several countries. DOACs are now the preferred anticoagulant of choice by many physicians given their favored properties compared with vitamin K antagonists. None of these DOACs have dose adjustments for patients with obesity in their product labels. The evidence surrounding the effectiveness of DOACs for the management of VTE in obese patients is thus limited. Our study suggests that the use of DOACs in obese patients is equally efficacious with similar six-month VTE recurrence rates in comparison with non-obese patients. This is one of the few retrospective studies that include a large patient cohort and the only study that uses the Health Facts Cerner National Data Warehouse.

There are no randomized controlled trials that have specifically investigated the effectiveness of DOAC use in the management of VTE in the obese population. However, several clinical trials included obese patients and performed a subgroup analysis demonstrating good efficacy [6-15]. These different trials mostly used a 100 kg cut-off while others also included a BMI of >35 as a cut-off [6,9]. The number of obese patients in these studies varied from a total of 85 to 3099 patients. The major phase III trials that reported weight‐based analyses demonstrated that the DOACs appeared to be equally efficacious in comparison with vitamin K agonists in the prevention of recurrent VTE in the highest‐weight category of each of the trials [6-15]. Furthermore, an abstract analyzed data from the EINSTEIN DVT/PE studies also found similar rates of VTE recurrence for patients in the highest weight category of > 100 kg [16].

The uncertainty about the effectiveness of DOACs in the management of VTE and atrial fibrillation stems from PK/PD studies. Given the lack of randomized controlled trials, PK/PD studies supplement data by analyzing the effects of body weight on half-lives, plasma drug concentrations, and expected drug exposure [4]. A study of the pharmacokinetics of apixaban included a comparison of patients with a weight of > 120 kg and BMI of ≥ 30 kg/m^2^ with a control group (weight of 65-85 kg) and found a shorter mean half‐life, 24% higher volume of distribution, 31% lower mean peak apixaban concentration, and 23% lower drug exposure in the high body weight group [17]. The authors concluded that there was no need to adjust the dose of apixaban in patients weighing > 120 kg because the effect was mild, and the clinical significance of these findings is to be confirmed with clinical studies [17]. In contrast, one small study on healthy volunteers examined the pharmacokinetics of rivaroxaban at the extremes of body weight and found similar half‐lives and peak plasma concentrations in patients weighing > 120 kg as compared with patients weighing 70-80 kg, suggesting that rivaroxaban might be the preferred DOAC in patients with obesity [18].

Limitations

We acknowledge certain limitations of this study. Given the lack of strong evidence, clinicians are reluctant to start DOAC therapy in patients with obesity, thus, only 13% of our patient cohort were in the group with body weight>120 Kg. Furthermore, though non-clinically significant, there was a higher proportion of females in the group with body weight<120 Kg, however, there were no significant differences in other baseline demographics thus controlling for possible contributing factors. Data were abstracted from the Health Facts Cerner National Data Warehouse and, therefore, the secondary outcomes or other patient-clinical or laboratory characteristics were limited and, consequently, lacking in our study. Last, while our study provides useful comparative data, the results are limited by the retrospective design. To be able to draw powerful conclusions, larger randomized controlled trials or multi-institutional prospective studies are imperative.· 

## Conclusions

This is one of the few retrospective studies with a large cohort evaluating the efficacy of DOAC therapy in patients with obesity. Our study suggests that the use of DOACs in obese patients is equally efficacious, with similar VTE recurrence rates in comparison with non-obese patients. Our results coincide with the subgroup analyses of other randomized controlled trials. However, our conclusion must be tempered given the lower proportion of patients with obesity. Therefore, further randomized controlled trials specifically addressing the efficacy of DOACs in the management of VTE in the obese population is pivotal to assist with clinical decision-making.
